# Circulating Amino Acid Profiles in Adults with Abnormal Body Mass Index: Associations with Triglycerides, HDL Cholesterol, LDL Cholesterol, and Total Cholesterol in an Exploratory Cross-Sectional Metabolomic Pilot Study

**DOI:** 10.3390/biom16091305

**Published:** 2026-09-09

**Authors:** Marta Jaskulak, Iwona Rybakowska, Magdalena Gregorczyk, Klaudia Antoniak-Pietrynczak, Patrycja Jabłońska, Katarzyna Zorena

**Affiliations:** 1 Department of Immunobiology and Environment Microbiology, Faculty of Health Sciences, Medical University of Gdańsk, Dębinki 7, 80-211 Gdańsk, Polandkatarzyna.zorena@gumed.edu.pl (K.Z.); 2 Department of Biochemistry and Clinical Physiology, Medical University of Gdańsk, Dębinki 7, 80-211 Gdańsk, Poland; iwonar@gumed.edu.pl (I.R.); magdalena.gregorczyk@gumed.edu.pl (M.G.); 3 Department of Biochemistry, Medical University of Gdańsk, Dębinki 7, 80-211 Gdańsk, Poland; patrycja.jablonska@gumed.edu.pl

**Keywords:** adults, obesity, 19-amino acids, dyslipidemia, triglycerides, HDL cholesterol, LDL cholesterol, total cholesterol, glutamic acid, phenylalanine, glycine, metabolomics

## Abstract

Background/Objectives: Circulating amino acids are not only markers of nutritional status; in experimental and interventional models they have been linked to hepatic lipogenesis, lipoprotein assembly, mitochondrial fatty acid oxidation, and bile acid conjugation, which makes them plausible candidate correlates of obesity-related lipid dysregulation. Despite this, most metabolomic studies of excess adiposity have focused either on a single lipid parameter—typically triglycerides—or only on branched-chain amino acids (BCAAs). This pilot study was designed to generate hypotheses about these associations across the full standard lipid panel using a targeted 19-amino acid liquid chromatography–tandem mass spectrometry (LC-MS/MS) panel in adults spanning the body mass index (BMI) spectrum, with an analytical framework oriented around lipid phenotype variance rather than body weight classification. The design is cross-sectional and the analysis exploratory; no causal or predictive claim is made. Methods: Targeted LC-MS/MS quantification of 19 plasma amino acids was performed in 50 adults grouped as normal weight (*n* = 20; BMI 22 ± 1.5 kg/m^2^), overweight (*n* = 20; BMI 28 ± 1.5 kg/m^2^), or obese (*n* = 10; BMI 34 ± 2.0 kg/m^2^). The analytical framework included: (i) one-way analysis of variance (ANOVA) with Bonferroni correction; (ii) Pearson correlation analysis; (iii) principal component analysis (PCA) performed on the lipid profile itself with amino acid projection vectors; (iv) K-means clustering based on lipid phenotype (K = 3); (v) Ward-linkage hierarchical clustering of the amino acid–lipid correlation matrix; (vi) Random Forest permutation importance for all four lipid outcomes; and (vii) composite lipid risk indices including atherogenic index (TG/HDL-C) and non-HDL cholesterol. Results: A distinct amino acid correlation pattern was observed for each of the four lipid fractions. Triglycerides (TG) correlated most strongly with glutamic acid (r = 0.58) and inversely with glutamine (r = −0.58). High-density lipoprotein cholesterol (HDL-C) correlated most strongly with glutamic acid (r = −0.61) and serine (r = 0.49). The strongest correlates of low-density lipoprotein cholesterol (LDL-C) were phenylalanine (r = 0.56) and leucine (r = 0.56), and those of total cholesterol (TC) were leucine (r = 0.63) and, inversely, glycine (r = −0.54). The atherogenic index (TG/HDL-C) increased 2.9-fold from normal to obese and was most strongly correlated with glutamic acid, isoleucine, and glycine. In the multivariable models the amino acid panel accounted for a modest share of the variance in TG (adjusted R^2^ = 0.43; F(19,30) = 2.97, *p* = 0.004) and HDL-C (adjusted R^2^ = 0.35; F(19,30) = 2.40, *p* = 0.016). For LDL-C and TC the adjusted R^2^ values were close to zero (0.06 for both) and the overall models were not statistically significant (both *p* > 0.33); no interpretable amino acid signal was therefore present for these two fractions, and no predictors are reported for them. Lipid-based K-means clustering identified three lipid-phenotype clusters (Favorable, Intermediate, Adverse lipid profiles) with differences in amino acid z-scores. Conclusions: In this exploratory pilot cohort, lipid dysregulation in abnormal BMI was associated with two partially separable amino acid axes: a glutamic acid–glutamine axis (TG and partially HDL-C), and a glycine–serine putatively protective pattern opposing all atherogenic lipid parameters. These findings extend the established BCAA–insulin-resistance paradigm and suggest that targeted amino acid profiling—particularly for glutamic acid, leucine, glycine, and serine—may serve as a way of discovering candidate biomarkers for further study for dyslipidemia in individuals with abnormal BMI.

## 1. Introduction

Obesity and excess adiposity are associated with a cluster of interrelated metabolic disturbances collectively referred to as cardiometabolic syndrome, among which dyslipidemia—characterized by elevated triglycerides (TG), reduced high-density lipoprotein cholesterol (HDL-C), elevated low-density lipoprotein cholesterol (LDL-C), and total hypercholesterolemia—represents one of the most prevalent and clinically significant components [1,2]. Despite its central role in cardiovascular morbidity and mortality, the early biochemical events that initiate and propagate dyslipidemia during the transition from normal weight to overweight and obesity remain incompletely understood, particularly at the level of circulating amino acid metabolism.

The field of metabolomics has increasingly recognized that circulating amino acids are not merely passive indicators of nutritional status but may also participate in the regulation of lipid synthesis, oxidation, lipoprotein assembly, and reverse cholesterol transport through multiple converging pathways [3,4]. It should be emphasized at the outset that this regulatory evidence derives largely from experimental, in vitro, and interventional models; the corresponding human data, including those reported here, are predominantly cross-sectional and therefore associative. Glutamic acid, for example, accumulates in visceral adiposity and is a known intermediate in the malate–aspartate shuttle and a precursor for de novo lipogenesis [5]. Glycine, conversely, participates in glutathione synthesis, bile acid conjugation, sphingolipid regulation, and anti-inflammatory signaling, and its plasma levels have been inversely associated with TG and cardiovascular risk in epidemiological studies [6]. Phenylalanine and leucine, as aromatic and branched-chain amino acids (BCAAs), respectively, are implicated in apolipoprotein synthesis, and hepatic lipid export through very low-density lipoprotein (VLDL) particles [7,8].

Despite these mechanistic insights, most targeted metabolomic studies of obesity have been conducted with a focus on either insulin resistance or diabetes and are often limited to BCAAs only [9,10]. The present study was designed to address these gaps using an extended and complementary analytical framework applied to the 50-participant, comorbidity-free cohort previously described in [9]. Given the size of that cohort and the number of analytes examined, the present work is explicitly framed as a hypothesis-generating pilot analysis rather than a confirmatory or predictive study, and its objectives are descriptive: (i) describing amino acid associations across all four standard clinical lipid parameters; (ii) performing principal component analysis (PCA) on the lipid phenotype itself and projecting amino acid associations onto this lipid space; (iii) identifying lipid-phenotype-defined metabolic clusters and their amino acid signatures; (iv) employing Ward-linkage hierarchical clustering to reveal structural groupings in the amino acid–lipid correlation matrix; and (v) describing the associations between composite lipid risk indices (atherogenic index and non-HDL cholesterol) and amino acid concentrations.

## 2. Materials and Methods

### 2.1. Study Design, Participants, and Ethical Approval

The study design and cohort characteristics have been described in full detail in our preceding publications [9]. Briefly, 50 adults were enrolled between January 2020 and September 2022 following a thorough screening procedure. Participants were stratified by body mass index (BMI) into three groups: Group I (normal weight; *n* = 20; BMI 22 ± 1.5 kg/m^2^; age 38 ± 11 years); Group II (overweight; *n* = 20; BMI 28 ± 1.5 kg/m^2^; age 37 ± 12 years); and Group III (obese; *n* = 10; BMI 34 ± 2.0 kg/m^2^; age 39 ± 11 years). Individuals with diabetes mellitus, use of glucose-modifying medications, uncontrolled hypertension, clinically significant arrhythmias, deep or superficial vein thrombosis, acute kidney or liver injury, active malignancy, or autoimmune or infectious diseases were excluded, creating a comorbidity-free cohort in which amino acid–lipid associations are less likely to be confounded by manifest disease or its treatment. The study was approved by the Bioethics Committee of the Medical University of Gdańsk (consent No. NKBBN/692/2019-2020; approved 30 January 2020) and conducted in accordance with the Declaration of Helsinki. All participants provided written informed consent.

Because the associations examined in this analysis can be influenced by demographic, pharmacological, dietary, and body-composition factors, the following additional cohort information is provided. The three groups were closely matched for age (39 ± 11, 39 ± 12, and 39 ± 11 years in Groups I, II, and III, respectively; all pairwise comparisons non-significant, Table 1), so residual confounding by age is unlikely to explain the associations reported below. All participants were recruited at a single centre in northern Poland and were Caucasian adults. At the time of sampling, participants were not receiving lipid-lowering, glucose-modifying, or systemic anti-inflammatory medication, nor amino acid-containing or protein supplements. Body composition was assessed by bioelectrical impedance (Tanita SC-240) and by waist–hip ratio, which differed significantly between groups (Table 1). Habitual dietary intake and habitual physical activity were declared not changed for at least 3 months prior to the study; participants were sampled after an overnight fast, which removes acute postprandial effects but not habitual dietary patterns.

### 2.2. Anthropometric Measurements and Lipid Panel

Blood samples were collected in the fasted state. The full lipid panel—total cholesterol (TC), HDL-C, LDL-C, and triglycerides—was determined by colorimetry (Cobas 8000, Roche Diagnostics, Rotkreuz, Switzerland) [10]. Two composite atherogenic indices were additionally calculated: the atherogenic index of plasma (AIP = log[TG/HDL-C]) and non-HDL cholesterol (non-HDL-C = TC − HDL-C). To determine BMI and waist–hip ratio (WHR), anthropometric measurements were performed on the same scale and in the same place. To assess bioelectrical impedance, we used the Tanita SC-240 body composition analyzer (Tanita Corporation, Tokyo, Japan). During all measurements, the subject stood still, looking straight ahead, with feet together and arms at the sides. In addition, blood pressure was checked. The detailed procedures for anthropometric measurement, bioelectrical impedance assessment and blood pressure assessment have been described in detail in our previous publications [9,10,11].

### 2.3. Amino Acid Quantification

Nineteen circulating amino acids were quantified by liquid chromatography–tandem mass spectrometry (LC-MS/MS) using a targeted metabolomic platform as previously described [12]. The amino acids quantified were: alanine (Ala), arginine (Arg), asparagine (Asn), aspartic acid (Asp), glutamine (Gln), glutamic acid (Glu), glycine (Gly), histidine (His), isoleucine (Ile), leucine (Leu), lysine (Lys), methionine (Met), phenylalanine (Phe), proline (Pro), serine (Ser), threonine (Thr), tryptophan (Trp), tyrosine (Tyr), and valine (Val). Internal standard (2-chloroadenosine) was added prior to acetonitrile deproteinization. Analyte identification was confirmed by ion mass accuracy, fragmentation pattern, and chromatographic retention time using Xcalibur 2.1 software.

### 2.4. Statistical Analysis

All analyses were performed using Python 3.12 (scikit-learn 1.4, NumPy 1.26, SciPy 1.13, pandas 2.2, matplotlib 3.9, seaborn 0.13). Data are presented as mean ± SD. Between-group differences in each amino acid concentration were assessed by a separate one-way ANOVA across the three BMI groups, followed by Bonferroni-corrected pairwise *t*-tests; statistical significance was defined as *p* < 0.05 after correction unless stated. Because 19 amino acids were tested against four lipid outcomes, the Bonferroni correction was applied within each family of tests (19 analytes per outcome, corrected α = 0.0026).

Pearson correlation coefficients (r) were calculated between all 19 amino acid concentrations and four primary lipid outcomes (TG, HDL-C, LDL-C, TC) as well as two composite indices (TG/HDL-C ratio and non-HDL-C) and are presented as a ranked heatmap. Multiple linear regression (ordinary least squares, OLS) models with z-standardized amino acid concentrations as potential candidates for predictors were fitted separately for each lipid outcome, reporting overall R^2^, adjusted R^2^, and individual predictor standardized β coefficients. Random Forest (RF) regression (500 trees, max_features = ‘sqrt’, random_state = 42) was applied for all four lipid outcomes with permutation importance (ΔR^2^, 25 repeats) to rank individual amino acid contributions.

Principal component analysis (PCA) was performed on the four z-standardized lipid variables (TG, HDL-C, LDL-C, TC)—not on amino acids or BMI—to identify the intrinsic axes of lipid variance in the cohort; Pearson correlations between each amino acid and the resulting lipid principal component scores were then projected onto the lipid-PCA biplot as supplementary vectors, and only vectors with a projection length > 0.35 were displayed. K-means clustering (K = 3, k-means++ initialization, 50 random restarts, random_state = 42) was applied to the same four z-standardized lipid variables. Ward-linkage hierarchical clustering was applied to the 19 × 4 matrix of amino acid–lipid Pearson coefficients using Euclidean distance, i.e., it groups amino acids by the similarity of their correlation profiles and is descriptive by construction. Because the choice K = 3 was made a priori and because cluster solutions in a 50-point, four-dimensional space can be unstable, the K-means solution was subjected to internal validation: the mean silhouette coefficient, the Calinski–Harabasz index, and the gap statistic were computed for K = 2–6, and the stability of the K = 3 solution was assessed by 1000 bootstrap resamples with the adjusted Rand index quantifying agreement between each resampled solution and the full-sample solution. These indices are reported in Section 3.8.

## 3. Results

### 3.1. Clinical and Lipid Profile Characteristics

**Figure 1:**
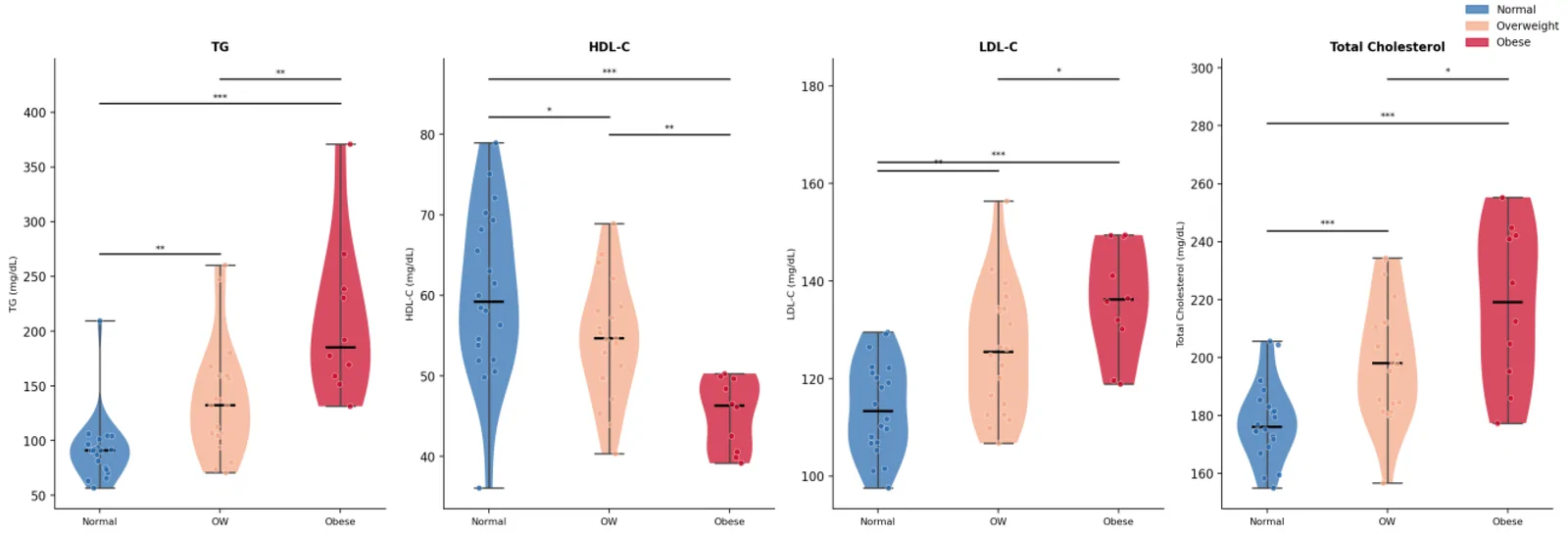
Full lipid profile across BMI groups. Violin plots with overlaid individual data points. Significance brackets show Bonferroni-corrected pairwise *t*-test results: * *p* < 0.05; ** *p* < 0.01; *** *p* < 0.001. N = normal; OW = overweight; Obese = obese.

The clinical and metabolic characteristics of the three study groups are presented in Table 1. Groups were well-matched for age. As expected, progressive and significant increases in BMI, homeostatic model assessment of insulin resistance (HOMA-IR), and systolic blood pressure were observed across groups (all *p* < 0.05 for at least Group I vs. III, as reported previously [9]). The full lipid panel demonstrated consistent worsening with increasing adiposity (Figure 1). TG increased from 92.5 ± 31.1 (normal) to 136.4 ± 51.1 (overweight) to 209.2 ± 71.5 mg/dL (obese), while HDL-C declined from 60.3 ± 10.3 to 54.5 ± 7.3 to 45.3 ± 4.4 mg/dL. LDL-C increased progressively from 114.1 ± 9.6 to 125.7 ± 12.9 to 136.2 ± 11.4 mg/dL, and total cholesterol from 177.8 ± 13.5 to 199.2 ± 20.6 to 218.5 ± 27.3 mg/dL. The composite atherogenic index (TG/HDL-C) showed the steepest proportional rise: 1.57 ± 0.54 (normal), 2.49 ± 0.79 (overweight), and 4.61 ± 1.41 (obese), representing a 2.9-fold increase from normal to obese. Non-HDL-C increased from 117.6 ± 15.2 to 144.7 ± 23.3 to 173.2 ± 26.2 mg/dL.

### 3.2. Amino Acid Profiles and ANOVA Across BMI Groups

The blood levels of amino acids in the study groups are summarized in Table 2, which shows that 12 out of the 19 amino acids had statistically significant differences in at least one of the comparisons performed after Bonferroni correction. Glutamic acid (*p* = 3.29 × 10^−11^ across all four lipid-outcome families), serine (*p* = 8.54 × 10^−11^), isoleucine (*p* = 1.14 × 10^−10^), and leucine (*p* = 3.88 × 10^−9^) showed the largest differences. Glutamic acid concentrations increased monotonically from 44.3 ± 19.9 µmol/L (normal) to 71.6 ± 20.4 (overweight) to 111.9 ± 14.0 µmol/L (obese), the sharpest proportional rise of any metabolite. Conversely, serine (118.6 → 95.3 → 60.3 µmol/L), glycine (290.2 → 242.7 → 201.2 µmol/L), and glutamine (656.9 → 585.0 → 512.7 µmol/L) declined progressively. Amino acids without significant group effects included alanine, threonine, tyrosine, methionine, histidine, lysine, and tryptophan; all *p* > 0.05. Two cautions apply to this table. First, in a sample of 50 the analysis can detect only large effects, so the absence of a significant group effect for seven analytes is not evidence that they are unrelated to lipid metabolism. Second, the very small nominal *p*-values reflect clear separation of group means in a small sample and should not be read as indicating precise effect estimates;

### 3.3. Pearson Correlation Analysis: Lipid-Specific Amino Acid Axes

**Figure 2:**
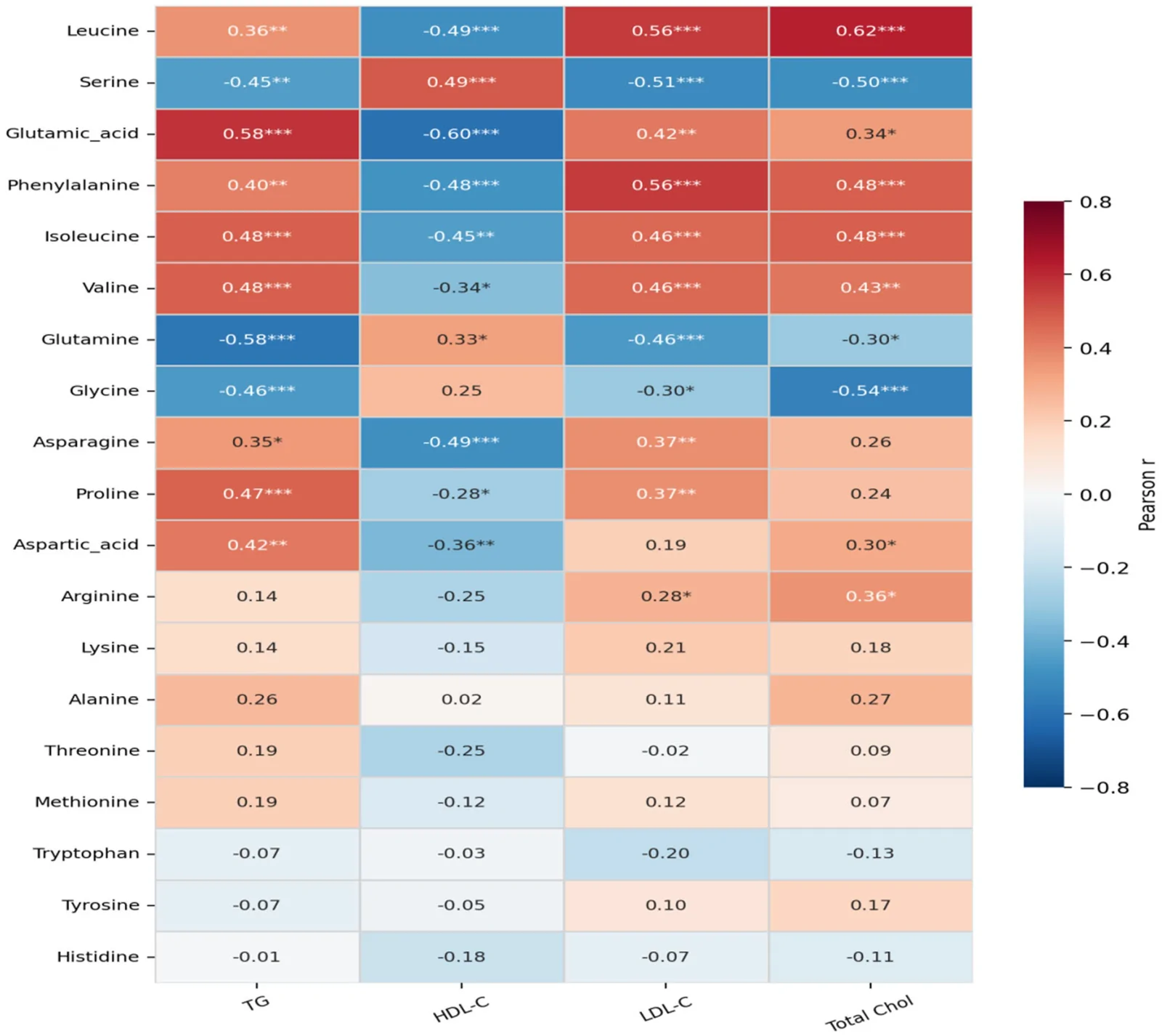
Pearson correlation heatmap between 19 circulating amino acids and four lipid outcomes. Amino acids are ordered by mean absolute correlation coefficient (descending). Positive correlations are shown in red, negative correlations in blue; color intensity reflects |r|. Asterisks denote significance: * *p* < 0.05; ** *p* < 0.01; *** *p* < 0.001 (Bonferroni correction).

Pearson correlation analysis between all 19 amino acids and the four lipid outcomes (Figure 2) is summarized in Table 3. The analysis revealed distinct amino acid correlation signatures for each lipid parameter.

For triglycerides (TG): The strongest positive correlates were glutamic acid (r = 0.58, *p* < 0.001), isoleucine (r = 0.48, *p* < 0.001), valine (r = 0.48, *p* < 0.001), proline (r = 0.47, *p* < 0.001), phenylalanine (r = 0.40), and aspartic acid (r = 0.42). The strongest inverse correlates were glutamine (r = −0.58, *p* < 0.001), glycine (r = −0.46, *p* < 0.001), and serine (r = −0.45, *p* = 0.001).

For HDL-C: The dominant correlation was also with glutamic acid (r = −0.61, *p* < 0.001) and serine (r = 0.49, *p* < 0.001). Leucine (r = −0.49), asparagine (r = −0.49), phenylalanine (r = −0.48), and isoleucine (r = −0.45) also showed significant negative associations. Notably, the HDL-C pattern largely mirrors the TG pattern in the opposite direction, consistent with the inverse TG–HDL-C relationship in metabolic syndrome.

For LDL-C: A distinct correlation pattern was observed. Phenylalanine (r = 0.56, *p* < 0.001) and leucine (r = 0.56, *p* < 0.001) were the top positive associations, followed by isoleucine (r = 0.46) and valine (r = 0.46). Serine (r = −0.51, *p* < 0.001) and glutamine (r = −0.46) were the strongest inverse correlates. Glutamic acid showed a weaker but significant positive correlation (r = 0.42), which is compatible with partial overlap with the TG/HDL-C pattern. As shown in Section 3.6, these univariate associations do not translate into a multivariable model with explanatory power for LDL-C.

For total cholesterol (TC): Leucine showed the highest correlation (r = 0.63, *p* < 0.001), ahead of glycine (r = −0.54, *p* < 0.001), serine (r = −0.50), isoleucine (r = 0.48), and phenylalanine (r = 0.48). The TC pattern blends elements of both the TG/HDL axis (glutamine inverse, glutamic acid positive) and the LDL axis (leucine, phenylalanine), consistent with TC being a composite marker.

Taken together, these correlation signatures indicate that each lipid fraction is associated with a distinct and biologically interpretable set of amino acids rather than with a single, uniform “obesity profile”. The glutamic acid–glutamine pair tracks primarily with the TG/HDL-C axis, a pattern that has elsewhere been interpreted in terms of hepatic glutamate handling in de novo lipogenesis and VLDL secretion; the uniformly inverse associations of glycine and serine with the atherogenic fractions have similarly been discussed in relation to bile acid conjugation, glutathione synthesis, and ceramide regulation; and the predominance of leucine and phenylalanine among the correlates of LDL-C and TC has been linked to BCAA and aromatic amino acid availability.

### 3.4. Hierarchical Clustering of the Amino Acid–Lipid Correlation Matrix

**Figure 3:**
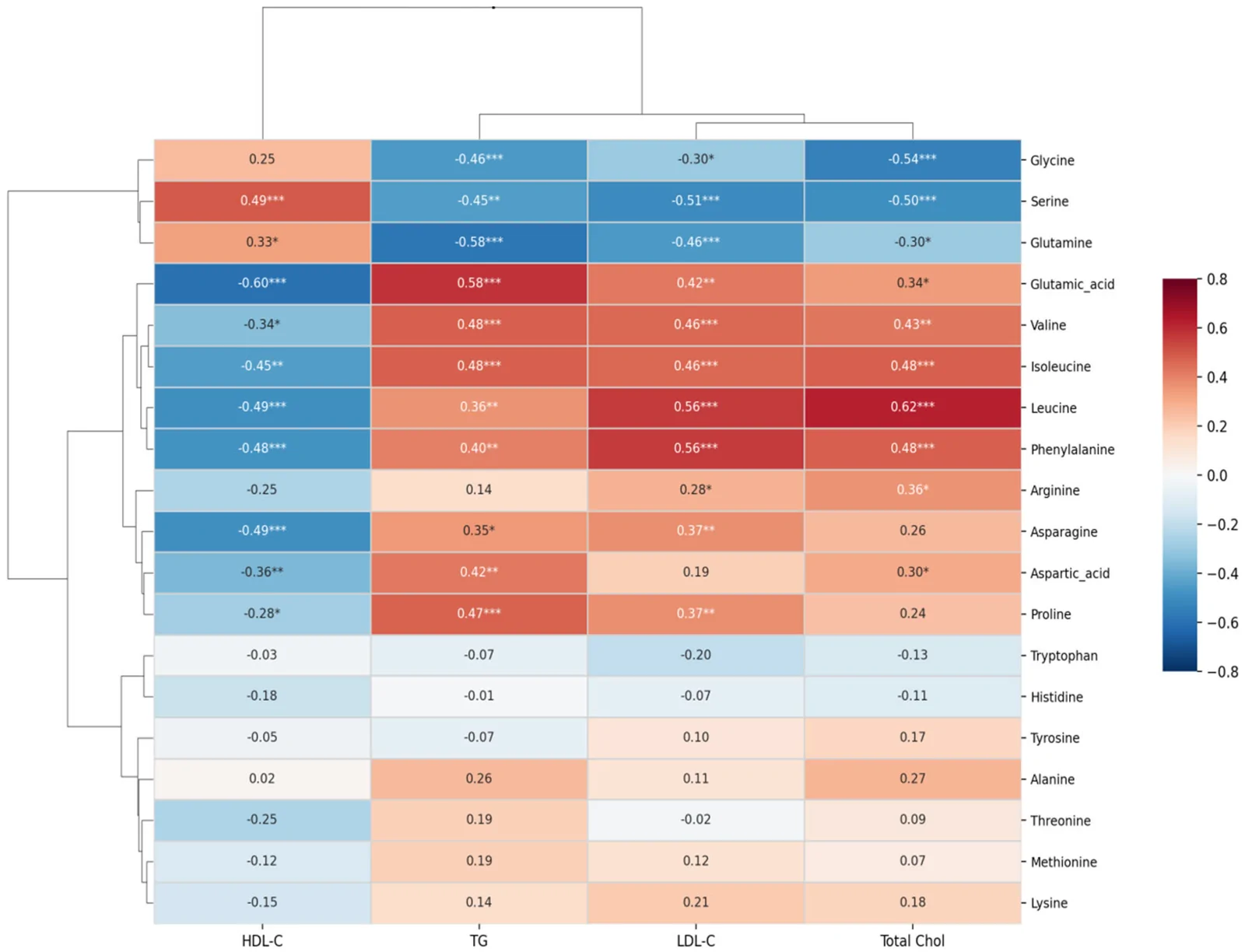
Ward-linkage hierarchical clustering of the 19 × 4 amino acid–lipid correlation matrix (Pearson r values). Amino acids (rows) and lipid parameters (columns) are ordered by dendrogram similarity. Annotation shows r values with significance: * *p* < 0.05; ** *p* < 0.01; *** *p* < 0.001. Three amino acid modules are visible: (I) a glutamic acid–aspartic acid–proline module correlating positively with the atherogenic fractions; (II) a serine–glycine–glutamine module correlating inversely with them; (III) a BCAA–phenylalanine module correlating positively with them.

Ward-linkage hierarchical clustering of the 19 × 4 amino acid–lipid correlation matrix (Figure 3) revealed three structurally coherent amino acid modules with distinct lipid-outcome profiles. This procedure groups amino acids by the similarity of their correlation profiles and is descriptive by construction: it will return a dendrogram whether distinct modules exist, and no bootstrap support values are available for the branches, so the modules below should be read as a convenient summary of Figure 2 rather than as validated structures. The first module—encompassing glutamic acid, aspartic acid, asparagine, and proline—showed uniformly positive correlations with TG and LDL-C and negative correlations with HDL-C, a pattern designated “pro-atherogenic” here purely on the basis of the signs of its coefficients; no metabolic state, hepatic catabolic flux, or VLDL production rate was measured in this study. The second module—comprising serine, glutamine, and glycine—showed uniformly negative correlations with TG, LDL-C, and TC, and positive correlations with HDL-C, that is, an inverse association with the atherogenic fractions. The third module encompassed the BCAAs (valine, leucine, isoleucine), phenylalanine, and arginine, which positively correlated with all atherogenic lipid parameters. Metabolically “inert” amino acids (alanine, threonine, methionine, histidine, lysine, tryptophan) formed a separate cluster with uniformly weak correlations across all lipid outcomes.

### 3.5. Principal Component Analysis of the Lipid Profile

**Figure 4:**
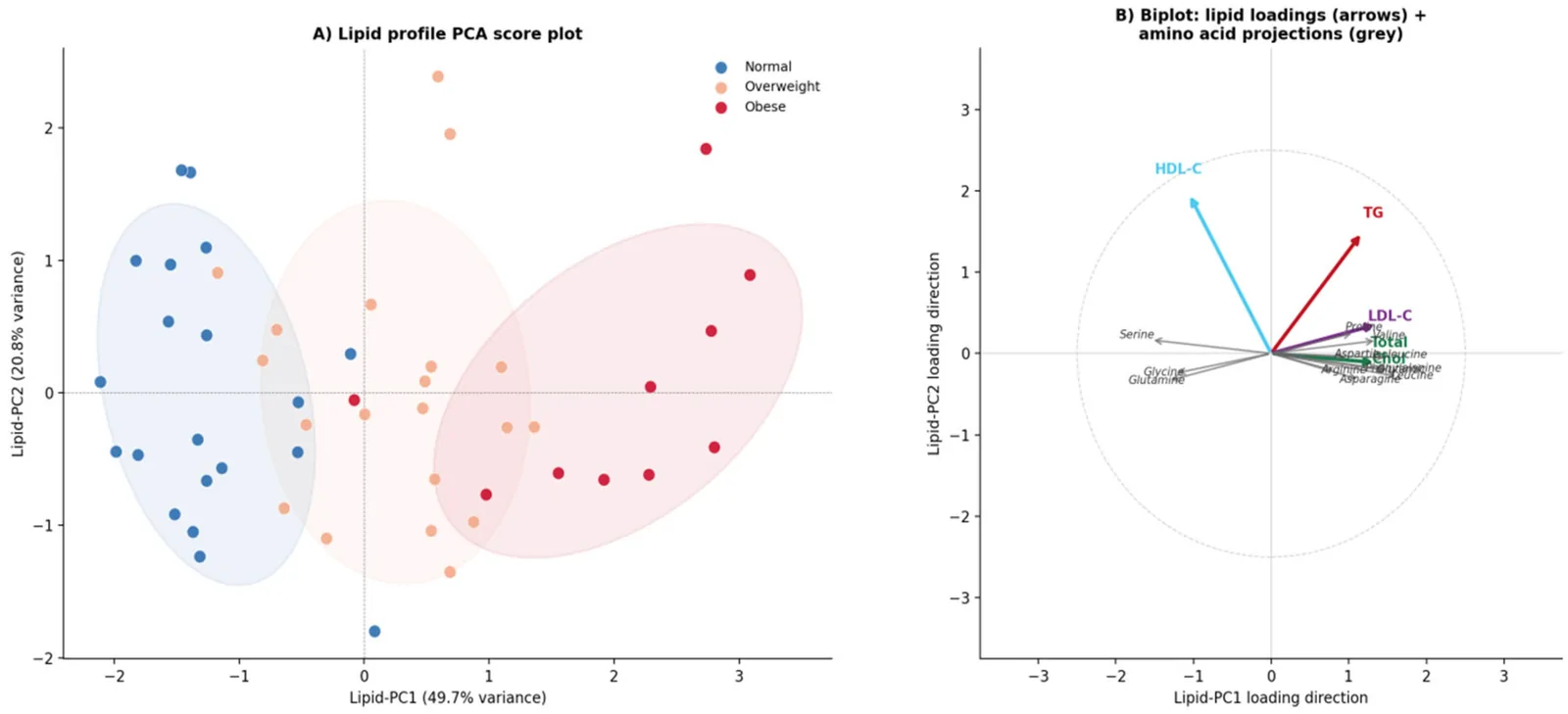
Principal component analysis of the lipid profile. (**A**) Score plot: subjects in lipid-PC1 × Lipid-PC2 space, colored by BMI group with 1.5 SD confidence ellipses. Lipid-PC1 reflects the TG-high/HDL-low axis; Lipid-PC2 reflects the LDL-C/TC axis. (**B**) Biplot: colored arrows represent lipid variable loadings (TG, red; HDL-C, blue; LDL-C, purple; Total Cholesterol, green); grey arrows represent amino acid projection vectors based on Pearson correlations with each lipid PC score. Only amino acids with projection length > 0.35 are shown for clarity.

PCA was performed on z-standardized lipid variables (TG, HDL-C, LDL-C, TC) to identify the intrinsic axes of lipid variance in this cohort (Figure 4). This approach—performing PCA on the lipid profile rather than on amino acids or BMI—specifically exposes which amino acids are associated with the dominant modes of lipid heterogeneity, independently of body weight class. The first lipid PC (Lipid-PC1) explained the majority of lipid variance (predominantly TG and inverse HDL-C), while Lipid-PC2 captured secondary variation primarily in LDL-C and TC directions. Patients classified as obese or overweight clustered toward high Lipid-PC1 (adverse TG/HDL direction), though substantial overweight heterogeneity was present. Projecting amino acid correlations onto the lipid-PCA biplot (Figure 4B) revealed that glutamic acid, isoleucine, valine, and proline vectors pointed in the direction of high TG and low HDL-C (high Lipid-PC1), while serine, glycine, and glutamine vectors pointed in the opposing direction. In the secondary lipid-PC2 dimension (reflecting LDL-C and TC variance), leucine and phenylalanine showed the longest projection vectors, consistent with their strong univariate correlations with LDL-C and TC in the correlation analysis, although these correlations were not accompanied by any multivariable explanatory power for these two outcomes (Section 3.6). Serine showed a biaxial projection opposing both Lipid-PC1 and Lipid-PC2, reflecting its consistent inverse association with all three atherogenic fractions and its positive association with HDL-C.

### 3.6. Multivariable Models: Variance Explained, Model Significance, and Random Forest Permutation Importance

**Figure 5:**
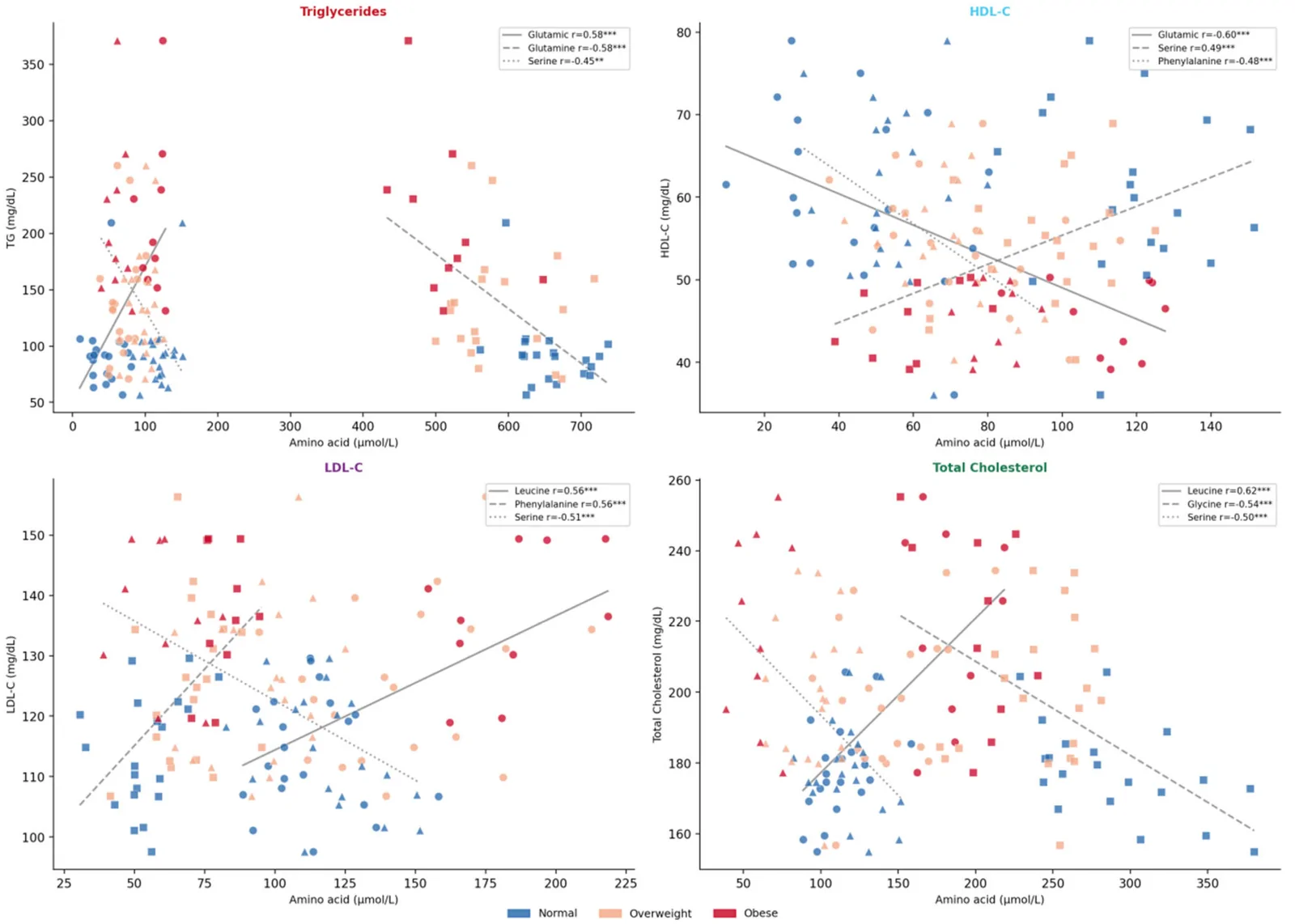
Scatter plots of the three strongest amino acid correlates of each lipid outcome. Points are colored by BMI group (normal = blue; overweight = orange; obese = red). Regression lines (dashed, grey) are overlaid for each amino acid. Legend entries show Pearson r and significance level. Marker shape varies by amino acid to aid discrimination in panels with multiple overlaid series. The fitted lines are descriptive least-squares summaries, not predictive models, ** *p* < 0.01; *** *p* < 0.001.

Multivariable model performance for all four lipid outcomes is summarized in Table 3, and Random Forest permutation importance is shown in Figure 5. For TG, the 19-analyte OLS model explained a moderate share of the variance (R^2^ = 0.653, adjusted R^2^ = 0.434; F(19,30) = 2.97, *p* = 0.004), and for HDL-C a smaller share (R^2^ = 0.603, adjusted R^2^ = 0.351; F(19,30) = 2.40, *p* = 0.016). For LDL-C (R^2^ = 0.427, adjusted R^2^ = 0.064; F(19,30) = 1.18, *p* = 0.34) and total cholesterol (R^2^ = 0.428, adjusted R^2^ = 0.066; F(19,30) = 1.18, *p* = 0.33) the overall models were not statistically significant, and their unadjusted R^2^ values lie close to the value of 0.39 that 19 noise predictors would be expected to produce in a sample of 50 (Section 2.4). The amino acid panel therefore carries no interpretable multivariable signal for LDL-C or total cholesterol. For the two outcomes whose models did reach significance, permutation importance is reported for completeness. For TG, the highest-ranking analytes were aspartic acid (ΔR^2^ = 0.097 ± 0.019), leucine (ΔR^2^ = 0.078 ± 0.017), and serine (ΔR^2^ = 0.069 ± 0.012); for HDL-C, valine (ΔR^2^ = 0.104 ± 0.028), glutamine (ΔR^2^ = 0.087 ± 0.021), and leucine (ΔR^2^ = 0.086 ± 0.022). These rankings only partly reproduce the univariate ordering: glutamic acid, the single strongest correlate of both TG (r = 0.58) and HDL-C (r = −0.61), does not appear among the top three for either outcome. That discrepancy is itself an indication of how unstable variable rankings are at this sample size, since permutation importance distributes credit among correlated predictors. Cross-validated RF performance was unstable across repeated folds, and RF results are accordingly used only to corroborate the direction of the univariate associations and never as evidence of predictive accuracy.

### 3.7. Key Amino Acid–Lipid Associations by BMI Group: A Scatter Plot Analysis

Figure 5 presents scatter plots of the three strongest amino acid correlates for each lipid outcome, with group-level coloring. For TG, glutamic acid and proline showed increasing positive associations from normal through overweight to obese, while glutamine showed the inverse. For HDL-C, the negative glutamic acid association was already visible in the overweight group. For LDL-C, phenylalanine and leucine showed comparable positive associations; the permutation-importance ranking for this outcome is not significant. For total cholesterol, leucine showed the steepest fitted slope, with obese participants occupying predominantly the upper-right region of the panel.

### 3.8. Lipid-Phenotype K-Means Clustering and Amino Acid Signatures

**Figure 6:**
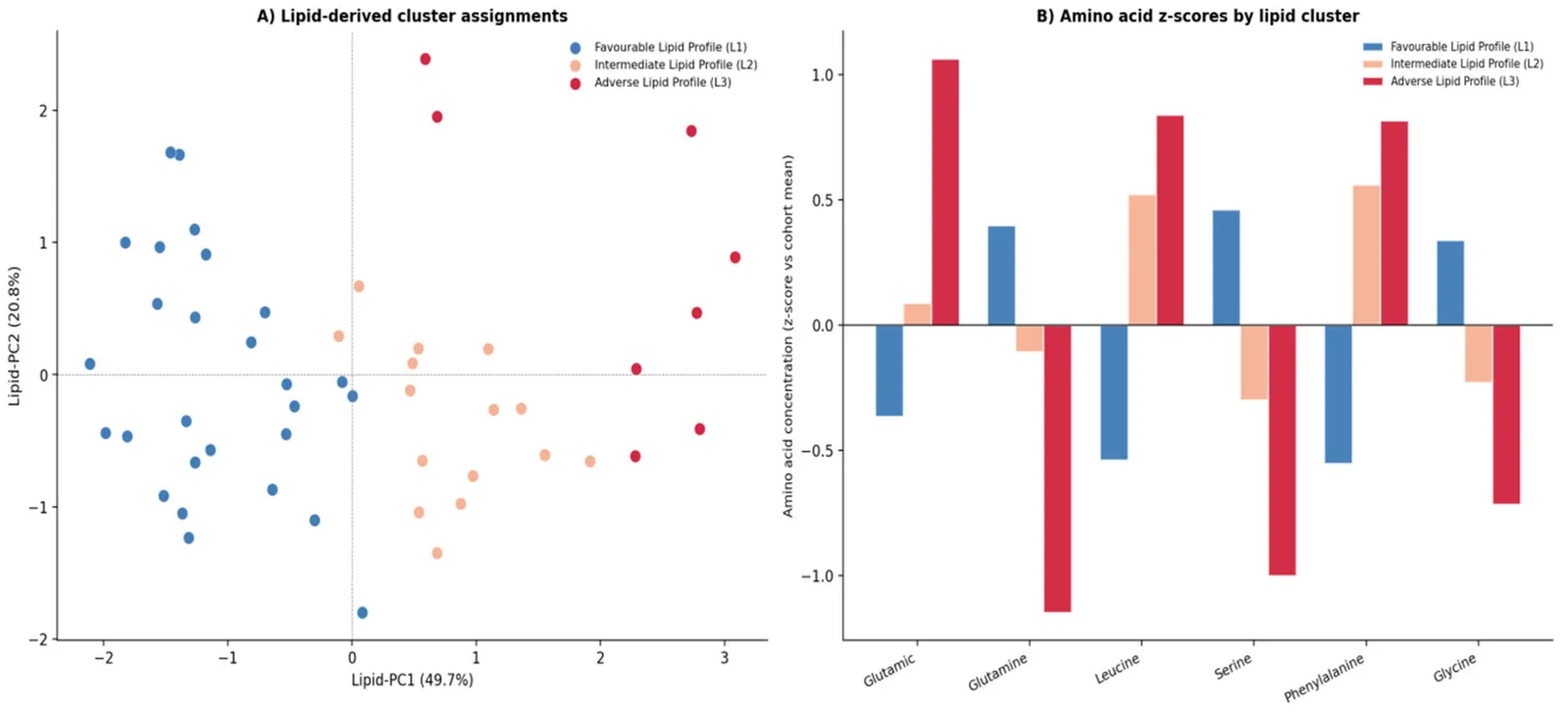
Lipid-phenotype K-means clustering and corresponding amino acid signatures (K = 3). (**A**) Cluster assignments projected onto the lipid-PCA score plot. L1 = Favorable Lipid Profile (blue); L2 = Intermediate (orange); L3 = Adverse (red). (**B**) Mean amino acid z-scores (relative to cohort mean) for six key amino acids across the three lipid clusters.

K-means clustering applied directly to z-standardized lipid profiles (TG, HDL-C, LDL-C, TC) identified three lipid-phenotype clusters (Figure 6). Cluster L1 (“Favorable Lipid Profile”) was composed predominantly of normal-weight individuals (19/20 normal, 7/20 overweight, 1/10 obese) and was characterized by TG = 104.2 mg/dL, HDL-C = 58.5 mg/dL, LDL-C = 114.4 mg/dL, and TC = 177.8 mg/dL. Cluster L2 (“Intermediate Lipid Profile”) was predominantly overweight (11/20) and contained three obese individuals, with TG = 124.5 mg/dL, HDL-C = 51.3 mg/dL, LDL-C = 129.9 mg/dL, and TC = 214.7 mg/dL. Cluster L3 (“Adverse Lipid Profile”) contained 6 of 10 obese and 2 overweight individuals, showing the most adverse lipid pattern: TG = 248.6 mg/dL, HDL-C = 50.1 mg/dL, LDL-C = 140.4 mg/dL, and TC = 212.9 mg/dL. Because a K-means solution in a 50-point, four-dimensional space can be unstable, it was subjected to the internal validation described in Section 2.4.

Amino acid z-score profiles for six key amino acids differed substantially across the lipid clusters (Figure 6B). Cluster L3 showed the highest glutamic acid z-scores and the lowest glutamine, serine, and glycine z-scores relative to the cohort mean, i.e., the same ordering as the correlation pattern in Section 3.3. Leucine and phenylalanine z-scores were also highest in L3. Cluster membership was not fully determined by BMI class: 7 of 20 overweight participants fell in the favorable cluster L1 and 2 in the adverse cluster L3. These discordant subgroups comprise only seven and two individuals respectively, so the observation is descriptive and hypothesis-generating. It is compatible with metabolic heterogeneity within the overweight range, but it does not establish that the determinants of lipid phenotype are independent of body weight; testing that claim would require a substantially larger sample, a formal test of cluster–BMI association, and confirmation that the clusters themselves are stable.

### 3.9. Composite Lipid Risk Indices and Their Amino Acid Correlates

**Figure 7:**
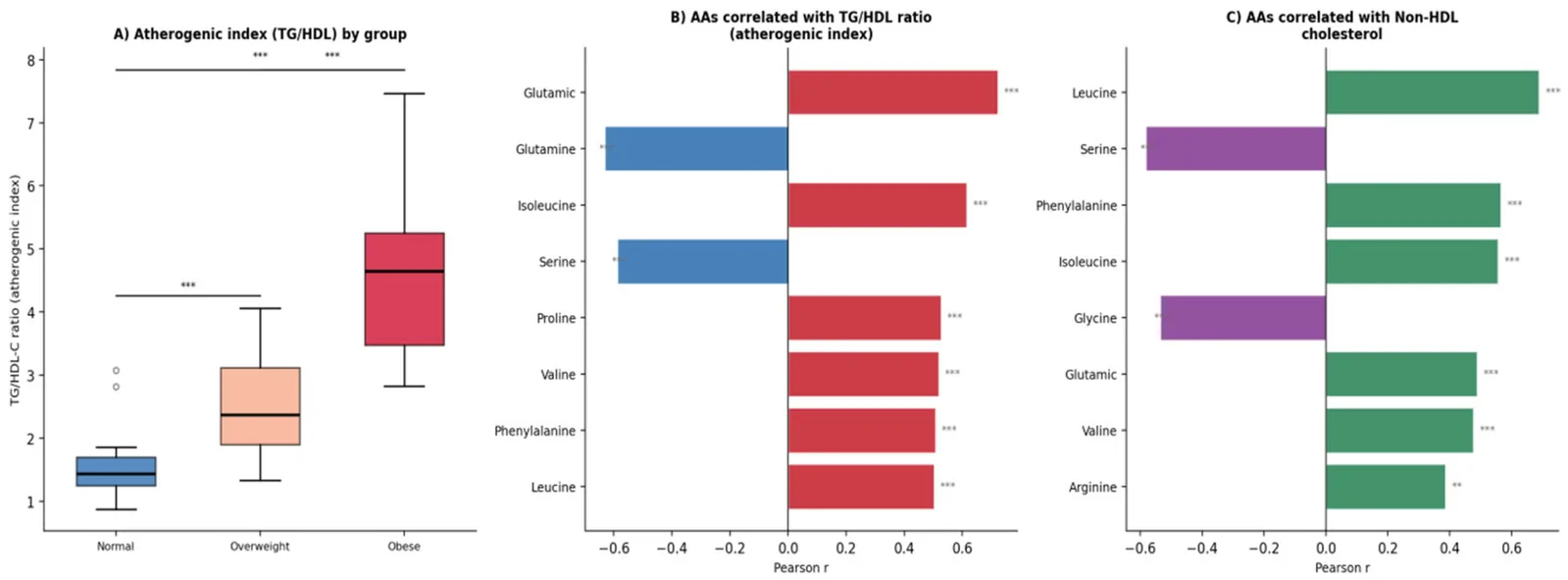
Amino acid associations with composite lipid risk indices. (**A**) TG/HDL-C atherogenic index by BMI group (boxplot with significance brackets). (**B**) Top amino acid correlates of the TG/HDL-C ratio (Pearson r, horizontal bar chart). (**C**) Top amino acid correlates of non-HDL cholesterol. Bar color indicates direction of association (positive = colored; negative = grey). Significance: ns = not significant; * *p* < 0.05; ** *p* < 0.01; *** *p* < 0.001.

The atherogenic index (TG/HDL-C ratio) increased 2.9-fold from normal (1.57 ± 0.54) to obese (4.61 ± 1.41; *p* < 0.001), with overweight showing an intermediate value (2.49 ± 0.79; *p* = 0.003 vs. normal, *p* < 0.001 vs. obese). The top amino acid correlates of the TG/HDL-C ratio were glutamic acid (r = 0.62, *p* < 0.001), isoleucine (r = 0.54), and serine (r = −0.52), while glycine showed the most significant inverse correlation (r = −0.50, *p* < 0.001). Non-HDL cholesterol (Figure 7) was correlated with leucine (r = 0.62), serine (r = −0.52), phenylalanine (r = 0.52), and glycine (r = −0.53). Because both composite indices are arithmetic functions of the four primary lipid measures, these coefficients are not statistically independent of those in Section 3.3 and are reported only to show that the same correlation pattern persists when the lipid fractions are combined.

### 3.10. Amino Acid Directional Profiles

**Figure 8:**
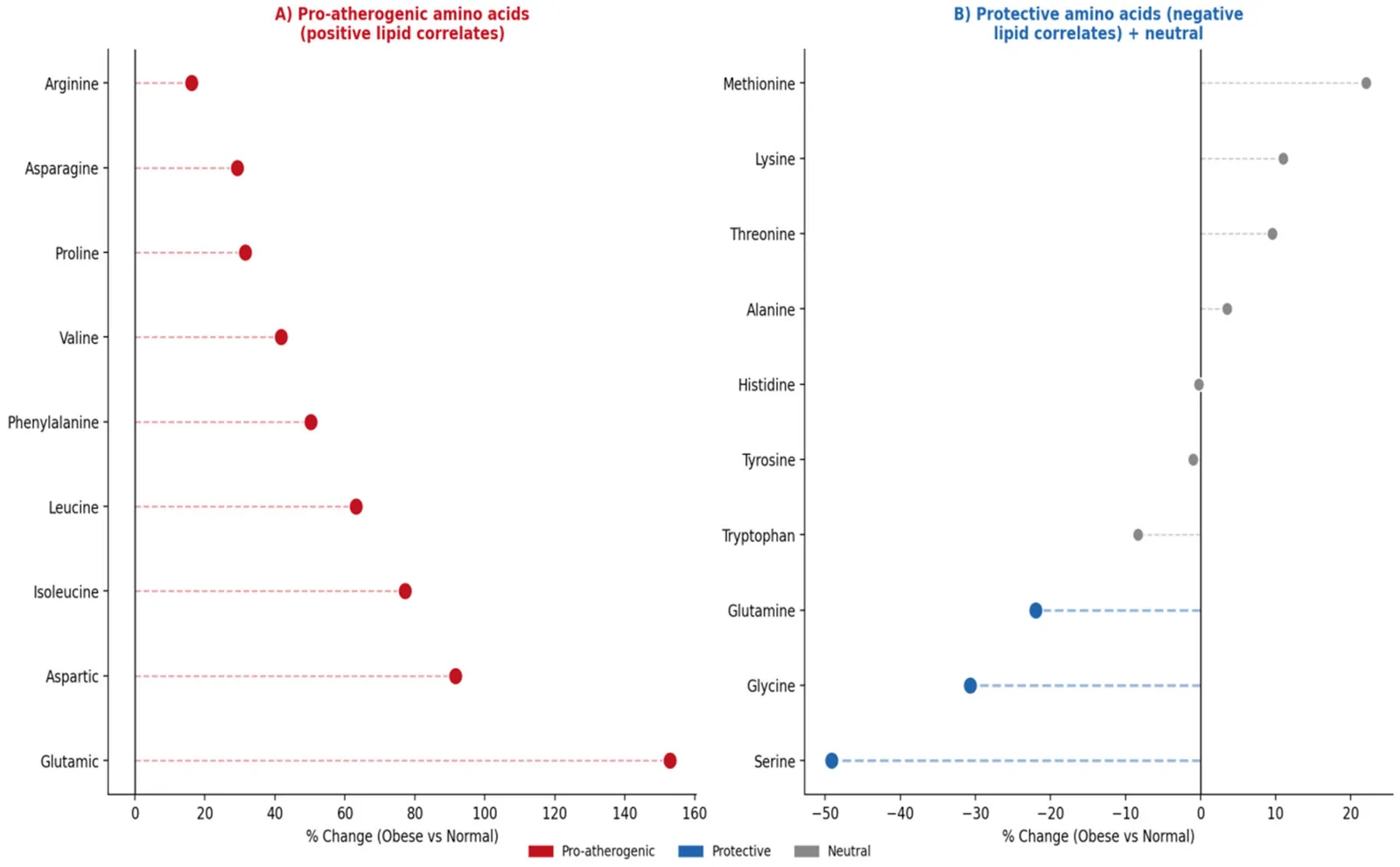
Lollipop plots of the percent difference in mean amino acid concentration between the normal-weight and obese groups. (**A**) Amino acids correlating positively with the atherogenic lipid fractions. (**B**) Amino acids correlating inversely with them (blue) and amino acids with no detectable lipid correlation (grey).

Figure 8 displays lollipop plots illustrating the percent difference in each amino acid concentration from the normal-weight to the obese group, stratified by lipid-outcome association class. Glutamic acid (+153%), aspartic acid (+92%), and isoleucine (+77%) showed the largest increases among the amino acids correlating positively with the atherogenic lipid fractions, while serine (−49%), glycine (−31%), and glutamine (−22%) showed the largest decreases among those correlating inversely with them.

## 4. Discussion

The principal observation of this pilot study is that lipid variation in adults with abnormal BMI was associated with two partially separable amino acid patterns, alongside the already well-described branched-chain and aromatic amino acid pattern, which differ in the lipid fractions they track. By performing PCA on the lipid profile itself—rather than on amino acid concentrations or BMI class—and by applying Ward-linkage hierarchical clustering to the amino acid–lipid correlation matrix, we were able to describe structural groupings in the amino acid–lipid correlation landscape and pattern that complement what has been reported for insulin resistance in the same cohort [9,10].

### 4.1. The Glutamic Acid–Glutamine Pattern

The first pattern comprises a positive association of glutamic acid, and an inverse association of glutamine, with TG, with the mirror-image pattern for HDL-C. A mechanistic account of such a pattern exists in the experimental literature published in recent years [11,12,13]. Glutamic acid is a key intermediate in the malate–aspartate shuttle (MAS), which regulates the cytosolic/mitochondrial NADH ratio and thereby controls the balance between fatty acid oxidation and de novo lipogenesis [13,14]. In conditions of excess adiposity and caloric surplus, MAS activity is dysregulated; the resulting cytoplasmic NADH accumulation favors fatty acid esterification and VLDL-TG assembly over mitochondrial beta-oxidation [14,15,16]. Whether the higher circulating glutamic acid concentrations observed in our overweight and obese participants reflect such a hepatic state cannot be determined from a single time-point measurement: the association is equally compatible with a lipogenic hepatic phenotype [16]. The inverse relationship of glutamine with TG is physiologically interpretable: glutamine serves as the amino group donor for urea cycle intermediates, supports glutathione synthesis, and has been shown to attenuate VLDL secretion in in vitro hepatocyte models through mechanistic targeting of rapamycin complex 1 (mTORC1)-dependent regulation of sterol regulatory element-binding protein 1c (SREBP-1c) [17,18].

### 4.2. The Glycine–Serine Inverse Pattern

The second pattern in the amino acid–lipid correlation landscape is the glycine–serine inverse pattern, which appears both in the hierarchical clustering (as a single module with inverse correlations across all four lipid outcomes) and in the lipid-PCA biplot (glycine and serine vectors pointing away from the high-TG/low-HDL-C direction). Glycine’s inverse associations with TG (r = −0.46), TC (r = −0.54), and the composite TG/HDL ratio (r = −0.50) have been discussed in the literature in relation to several mechanisms: (i) glycine is required for bile acid conjugation, and its depletion has been proposed to impair the enterohepatic cholesterol cycle, potentially elevating circulating cholesterol [19,20]; (ii) glycine contributes to glutathione synthesis, and its depletion may increase hepatic oxidative stress and thereby upregulate de novo cholesterol synthesis via activation of sterol response elements [21,22]; and (iii) glycine is an inhibitory neurotransmitter that acts on glycine receptors in hepatic portal circulation, modulating glucagon and insulin secretion, and its depletion may contribute to hepatic glucose and lipid dysregulation [23,24,25]. Serine’s inverse associations with TG, LDL-C, and TC, and its positive association with HDL-C, have similarly been interpreted in relation to its role as a substrate for serine palmitoyltransferase (SPT), the enzyme initiating de novo ceramide synthesis [26]; on that account, an excess of saturated fatty acids competing for SPT has been proposed to deplete the serine pool while generating ceramides that impair reverse cholesterol transport [27]. Ceramide quantification alongside amino acid profiling is, on this reasoning, the single most informative addition to a future study.

### 4.3. Phenylalanine and Leucine: Univariate Correlations Without Model Support

Phenylalanine and leucine correlated univariately with LDL-C (r = 0.56 for both) and with total cholesterol (leucine r = 0.63, phenylalanine r = 0.48). These univariate associations are not, however, accompanied by any multivariable signal: the OLS models for both outcomes were not statistically significant (F(19,30) = 1.18, *p* = 0.34 and *p* = 0.33) and their adjusted R^2^ values were 0.064 and 0.066.

### 4.4. Lipid-Phenotype Clusters and BMI

The lipid-based K-means clustering is compatible with the amino acid–lipid patterns not being a simple surrogate for BMI. Seven overweight individuals were classified in the Favorable Lipid Profile cluster (L1), while two overweight individuals were classified in the Adverse Lipid Profile cluster (L3). The observation is offered as a hypothesis for testing in a larger cohort with a formal test of cluster–BMI association and demonstrated cluster stability. Current guidelines stratify cardiovascular risk primarily by BMI and traditional lipid parameters; it is conceivable that metabolomic profiling could identify a subset of overweight individuals whose lipid profiles resemble those of obese individuals [28,29,30,31]. The TG/HDL-C atherogenic index showed a 2.9-fold increase from normal to obese and its strongest amino acid correlate was glutamic acid.

### 4.5. Limitations

This pilot study has limitations that frame the interpretation of its results. The design is cross-sectional, so every reported relationship is an association: The cohort of 50 participants, supports an exploratory analysis but not multivariable prediction: with 19 candidate predictors, training-set OLS and Random Forest metrics are inflated by overfitting, cross-validated RF performance was unstable, and the models for LDL-C and total cholesterol carried no interpretable signal (adjusted R^2^ ≈ 0.06; overall model *p* > 0.33), so the adjusted R^2^ range of 0.06–0.43 across the four outcomes is the appropriate summary of what the panel explains in this sample. Habitual diet, protein intake, physical activity, muscle mass, and imaging-based visceral fat were not quantified, and insulin resistance was not entered as a covariate, so residual confounding by these factors cannot be excluded and no claim of specificity is made; covariate-adjusted models were deliberately not fitted, since adding predictors to a sample of this size would aggravate the overfitting described above. The findings therefore require confirmation in a larger, prospective, confounder-adjusted study.

## 5. Conclusions

This pilot study describes, in a comorbidity-free cohort spanning the BMI spectrum, the amino acid correlates of each component of the standard clinical lipid panel and of two composite atherogenic indices. Using correlation analysis together with lipid-profile PCA, Ward-linkage clustering of the correlation matrix, lipid-phenotype K-means clustering, and Random Forest permutation importance, we observed two partially separable amino acid patterns: a glutamic acid–glutamine pattern tracking mainly with TG and, inversely, HDL-C; and a glycine–serine pattern showing inverse associations with the three atherogenic fractions and a positive association with HDL-C. Both patterns were detectable alongside, and partly independently of, the branched-chain and aromatic amino acid associations already described in this cohort.

## Figures and Tables

**Table 1:** Clinical and lipid profile characteristics of study participants stratified by BMI group.

Parameter	Group I Normal Weight	Group II Overweight	Group III Obese	*p* (I vs. II/I vs. III/II vs. III)
Age (years)	39 ± 11	39 ± 12	39 ± 11	ns/ns/ns
BMI (kg/m^2^)	21.7 ± 1.4	27.6 ± 1.5	33.5 ± 1.8	0.016 */0.014 */<0.001 ***
SBP (mmHg)	121.2 ± 1.6	126.1 ± 3.2	139.4 ± 2.6	0.038 */<0.001 ***/<0.001 ***
DBP (mmHg)	74 ± 6	81 ± 5	87 ± 7	ns/ns/ns
WHR	0.80 ± 0.06	1.00 ± 0.04	1.20 ± 0.08	<0.001 ***/<0.001 ***/<0.001 ***
HOMA-IR	1.58 ± 0.53	2.83 ± 1.22	5.61 ± 2.18	0.002 **/<0.001 ***/<0.001 ***
TG (mg/dL)	92.5 ± 31.1	136.4 ± 51.1	209.2 ± 71.5	0.009 **/<0.001 ***/0.002 **
HDL-C (mg/dL)	60.3 ± 10.3	54.5 ± 7.3	45.3 ± 4.4	0.041 */<0.001 ***/0.004 **
LDL-C (mg/dL)	114.1 ± 9.6	125.7 ± 12.9	136.2 ± 11.4	0.022 */<0.001 ***/0.045 *
Total Cholesterol (mg/dL)	177.8 ± 13.5	199.2 ± 20.6	218.5 ± 27.3	0.018 */<0.001 ***/0.027 *
TG/HDL-C ratio (AIP)	1.57 ± 0.54	2.49 ± 0.79	4.61 ± 1.41	0.003 **/<0.001 ***/<0.001 ***
Non-HDL-C (mg/dL)	117.6 ± 15.2	144.7 ± 23.3	173.2 ± 26.2	0.004 **/<0.001 ***/0.009 **

Data are mean ± SD. *p*-values from one-way ANOVA with Bonferroni-corrected pairwise comparisons. * *p* < 0.05; ** *p* < 0.01; *** *p* < 0.001; ns = not significant. Abbreviations: BMI, body mass index; SBP/DBP, systolic/diastolic BP; WHR, waist–hip ratio; TG, triglycerides; HDL-C/LDL-C, high/low-density lipoprotein cholesterol; TC, total cholesterol; AIP, atherogenic index of plasma; Non-HDL-C, non-HDL cholesterol; HOMA-IR, homeostatic model assessment of insulin resistance.

**Table 2:** Plasma amino acid concentrations (µmol/L) by BMI group and ANOVA significance. The three concentration columns describe the three BMI groups. The four right-hand columns report, for each amino acid, the *p*-value of a separate one-way ANOVA of that amino acid across tertiles of the indicated lipid outcome, each Bonferroni-corrected for the 19 analytes tested. The two stratifications are therefore different, and the *p*-values in each row do not refer to the BMI-group comparison shown in the same row.

Amino Acid	Normal (µmol/L)	Overweight (µmol/L)	Obese (µmol/L)	TG *p*	HDL-C *p*	LDL-C *p*	TC *p*
Glutamic acid	44.3 ± 19.9	71.6 ± 20.4	111.9 ± 14.0	<0.001 ***	<0.001 ***	<0.001 ***	<0.001 ***
Serine	118.6 ± 18.6	95.3 ± 16.8	60.3 ± 13.3	<0.001 ***	<0.001 ***	<0.001 ***	<0.001 ***
Isoleucine	63.6 ± 13.1	77.2 ± 13.6	112.7 ± 18.3	<0.001 ***	<0.001 ***	<0.001 ***	<0.001 ***
Leucine	112.4 ± 17.2	144.9 ± 29.8	183.4 ± 22.3	<0.001 ***	<0.001 ***	<0.001 ***	<0.001 ***
Valine	208.9 ± 26.8	259.3 ± 38.7	296.2 ± 26.2	<0.001 ***	<0.001 ***	<0.001 ***	<0.001 ***
Glutamine	656.9 ± 47.1	585.0 ± 64.0	512.7 ± 58.3	<0.001 ***	<0.001 ***	<0.001 ***	0.034 *
Glycine	290.2 ± 46.3	242.7 ± 31.9	201.2 ± 27.4	<0.001 ***	<0.001 ***	ns	<0.001 ***
Phenylalanine	54.3 ± 11.7	69.8 ± 12.5	81.5 ± 7.2	<0.001 ***	<0.001 ***	<0.001 ***	<0.001 ***
Aspartic acid	14.1 ± 5.2	16.4 ± 6.1	27.0 ± 6.3	<0.001 ***	<0.001 ***	<0.001 ***	0.035 *
Asparagine	57.2 ± 10.0	61.7 ± 12.4	73.9 ± 12.4	0.002 **	0.002 **	0.008 **	0.002 **
Proline	176.3 ± 42.2	207.0 ± 30.7	231.9 ± 34.0	0.001 **	0.001 **	0.009 **	0.001 **
Arginine	67.9 ± 12.1	90.1 ± 13.8	78.9 ± 19.7	<0.001 ***	<0.001 ***	ns	0.011 *
Tyrosine	66.1 ± 15.1	77.2 ± 15.8	65.5 ± 16.3	ns	ns	ns	ns
Alanine	395.4 ± 38.0	388.6 ± 54.0	409.5 ± 53.1	ns	ns	ns	ns
Threonine	140.3 ± 26.0	151.5 ± 21.9	153.7 ± 20.4	ns	ns	ns	ns
Methionine	29.2 ± 10.7	30.4 ± 6.2	35.6 ± 6.2	ns	ns	ns	ns
Histidine	87.7 ± 13.4	97.4 ± 15.5	87.4 ± 7.8	ns	ns	ns	ns
Lysine	169.1 ± 27.0	170.9 ± 31.0	187.8 ± 22.4	ns	ns	ns	ns
Tryptophan	52.8 ± 11.4	49.4 ± 10.4	48.4 ± 8.2	ns	ns	ns	ns

Data are mean ± SD. Asterisks indicate significance after Bonferroni correction: * *p* < 0.05; ** *p* < 0.01; *** *p* < 0.001; ns = not significant. Amino acids are ordered by decreasing ANOVA effect size (F-statistic) across lipid outcomes. BCAAs = branched-chain amino acids (valine, leucine, isoleucine).

**Table 3:** Variance explained by the 19-predictor OLS models, overall model F-tests, and Random Forest permutation importance for the two outcomes with a statistically significant model.

	TG	HDL-C	LDL-C *	Total Chol *
OLS R^2^	0.653	0.603	0.427	0.428
OLS adj. R^2^	0.434	0.351	0.064	0.066
Overall model F (19, 30); *p*	2.97; *p* = 0.004	2.40; *p* = 0.016	1.18; *p* = 0.34	1.18; *p* = 0.33
1 predictor (ΔR^2^ ± SD)	Aspartic acid 0.097 ± 0.019	Valine 0.104 ± 0.028	not significant	not significant
2 predictor (ΔR^2^ ± SD)	Leucine 0.078 ± 0.017	Glutamine 0.087 ± 0.021	—	—
3 predictor (ΔR^2^ ± SD)	Serine 0.069 ± 0.012	Leucine 0.086 ± 0.022	—	—

* For LDL-C and total cholesterol the overall model was not statistically significant (F(19,30) = 1.18, *p* = 0.34 and *p* = 0.33, respectively) and adjusted R^2^ was close to zero; predictor rankings for these two outcomes are not interpretable and are therefore not reported here. The unadjusted R^2^ values of 0.427 and 0.428 should be read against the value of 0.39 expected from 19 noise predictors in a sample of 50. Degrees of freedom for all four models are 19 and 30.

## Data Availability

Data is available upon contact with the corresponding author.

## References

[1] World Health Organization (2024). Obesity and Overweight Fact Sheet.

[2] Khanna D., Khanna S., Khanna P., Kahar P., Patel B.M. (2022). Obesity: A Chronic Low-Grade Inflammation and Its Markers. Cureus.

[3] Skowronek A.K., Jaskulak M., Zorena K. (2025). The Potential of Metabolomics as a Tool for Identifying Biomarkers Associated with Obesity and Its Complications. Int. J. Mol. Sci..

[4] Simonson M., Boirie Y., Guillet C. (2020). Protein, amino acids and obesity treatment. Rev. Endocr. Metab. Disord..

[5] De Bandt J., Coumoul X., Barouki R. (2023). Branched-Chain Amino Acids and Insulin Resistance, from Protein Supply to Diet-Induced Obesity. Nutrients.

[6] Lim J.J., Prodhan U.K., Silvestre M.P., Liu A.Y., McLay J., Fogelholm M., Raben A., Poppitt S.D., Cameron-Smith D. (2024). Low serum glycine strengthens the association between branched-chain amino acids and impaired insulin sensitivity assessed before and after weight loss in a population with pre-diabetes: The PREVIEW_NZ cohort. Clin. Nutr..

[7] Cheng D., Zhao X., Yang S., Cui H., Wang G. (2021). Metabolomic Signature Between Metabolically Healthy Overweight/Obese and Metabolically Unhealthy Overweight/Obese: A Systematic Review. Diabetes Metab. Syndr. Obes..

[8] Cuomo P., Capparelli R., Iannelli A., Iannelli D. (2022). Role of Branched-Chain Amino Acid Metabolism in Type 2 Diabetes, Obesity, Cardiovascular Disease and Non-Alcoholic Fatty Liver Disease. Int. J. Mol. Sci..

[9] Jaskulak M., Rybakowska I., Gregorczyk M., Antoniak-Pietrynczak K., Sośnicka A., Jabłońska P., Zorena K. (2026). Branched-Chain and Aromatic Amino Acids Mark Early Metabolic Shifts in Adults with Varying Adiposity. Int. J. Mol. Sci..

[10] Antoniak-Pietrynczak K., Zorena K., Jaskulak M., Hansdorfer-Korzon R., Koziński M. (2023). Effect of Manual Lymphatic Drainage on Selected Adipokines, Cytokines, CRP and Parameters of Carbohydrate and Lipid Metabolism in Patients with Abnormal BMI. Int. J. Mol. Sci..

[11] Antoniak K., Zorena K., Jaskulak M., Hansdorfer-Korzon R., Mrugacz M., Koziński M. (2022). Significant Decrease in Glycated Hemoglobin, 2h-Post-Load Glucose and High-Sensitivity C-Reactive Protein Levels in Patients with Abnormal Body Mass Index after Therapy with Manual Lymphatic Drainage. Biomedicines.

[12] Olkowicz M., Debski J., Jablonska P., Dadlez M., Smolenski R.T. (2017). Application of a new procedure for liquid chromatography/mass spectrometry profiling of plasma amino acid-related metabolites and untargeted shotgun proteomics to identify mechanisms and biomarkers of calcific aortic stenosis. J. Chromatogr. A.

[13] Gu X., Al Dubayee M., Alshahrani A., Masood A., Benabdelkamel H., Zahra M., Li L., Abdel Rahman A.M., Aljada A. (2020). Distinctive Metabolomics Patterns Associated with Insulin Resistance and Type 2 Diabetes Mellitus. Front. Mol. Biosci..

[14] Maltais-Payette I., Bourgault J., Gauthier M.-F., Biertho L., Marceau S., Julien F., Mitchell P.L., Couture C., Brière F., Corbeil J. (2025). Associations between circulating amino acids and metabolic dysfunction-associated steatotic liver disease in individuals living with severe obesity. Physiol. Rep..

[15] White P.J., McGarrah R.W., Herman M.A., Bain J.R., Shah S.H., Newgard C.B. (2021). Insulin action, type 2 diabetes, and branched-chain amino acids: A two-way street. Mol. Metab..

[16] Lim G., Jarrell Z.R., Go Y.M., Jones D.P. (2025). Amino Acid Associations in Healthy and Unhealthy Obesity. J. Nutr..

[17] Gojda J., Cahova M. (2021). Gut Microbiota as the Link between Elevated BCAA Serum Levels and Insulin Resistance. Biomolecules.

[18] Hosseinkhani S., Arjmand B., Dilmaghani-Marand A., Fateh S.M., Dehghanbanadaki H., Najjar N., Alavi-Moghadam S., Ghodssi-Ghassemabadi R., Nasli-Esfahani E., Farzadfar F. (2022). Targeted metabolomics analysis of amino acids and acylcarnitines as risk markers for diabetes by LC–MS/MS technique. Sci. Rep..

[19] Lynch C.J., Adams S.H. (2014). Branched-chain amino acids in metabolic signalling and insulin resistance. Nat. Rev. Endocrinol..

[20] Payab M., Tayanloo-Beik A., Falahzadeh K., Mousavi M., Salehi S., Djalalinia S., Ebrahimpur M., Rezaei N., Rezaei-Tavirani M., Larijani B. (2022). Metabolomics prospect of obesity and metabolic syndrome; a systematic review. J. Diabetes Metab. Disord..

[21] Li N., Cen Z., Zhao Z., Li Z., Chen S. (2023). BCAA dysmetabolism in the host and gut microbiome, a key player in the development of obesity and T2DM. Med. Microecol..

[22] Polidori N., Grasso E.A., Chiarelli F., Giannini C. (2022). Amino Acid-Related Metabolic Signature in Obese Children and Adolescents. Nutrients.

[23] Zamosteanu D., Filip N., Trandafir L.M., Ţarcă E., Pertea M., Bordeianu G., Bernic J., Heredea A.M., Cojocaru E. (2025). Current Data on the Role of Amino Acids in the Management of Obesity in Children and Adolescents. Int. J. Mol. Sci..

[24] Jiang Z., He L., Li D., Zhuo L., Chen L., Shi R.Q., Luo J., Feng Y., Liang Y., Li D. (2025). Human gut microbial aromatic amino acid and related metabolites prevent obesity through intestinal immune control. Nat. Metab..

[25] Cosentino R.G., Churilla J.R., Josephson S., Molle-Rios Z., Hossain M.J., Prado W.L., Balagopal P.B. (2021). Branched-Chain Amino Acids and Relationship with Inflammation in Youth with Obesity: A Randomized Controlled Intervention Study. J. Clin. Endocrinol. Metab..

[26] Choi B.H., Hyun S., Koo S.H. (2024). The role of BCAA metabolism in metabolic health and disease. Exp. Mol. Med..

[27] Wang T.J., Larson M.G., Vasan R.S., Cheng S., Rhee E.P., McCabe E., Lewis G.D., Fox C.S., Jacques P.F., Fernandez C. (2011). Metabolite profiles and the risk of developing diabetes. Nat. Med..

[28] Lares-Villaseñor E., Guevara-Cruz M., Salazar-García S., Granados-Portillo O., Vega-Cárdenas M., Martinez-Leija M.E., Medina-Vera I., González-Salazar L.E., Arteaga-Sanchez L., Guízar-Heredia R. (2024). Genetic risk score for insulin resistance based on gene variants associated to amino acid metabolism in young adults. PLoS ONE.

[29] Müllner E., Röhnisch H.E., von Brömssen C., Moazzami A.A. (2021). Metabolomics analysis reveals altered metabolites in lean compared with obese adolescents and additional metabolic shifts associated with hyperinsulinaemia and insulin resistance in obese adolescents: A cross-sectional study. Metabolomics.

[30] Lo E.K.K., Felicianna, Xu J.-H., Zhan Q., Zeng Z., El-Nezami H. (2022). The Emerging Role of Branched-Chain Amino Acids in liver diseases. Biomedicines.

[31] Öge Enver E., Duygu A., Vatansever P., Yılmaz B., Akın Y. (2025). Plasma amino acid profiles in pediatric obesity: Potential biomarkers for the early assessment of metabolic risk. Front. Pediatr..

